# Lessons from a one-year hospital-based surveillance of acute respiratory infections in Berlin- comparing case definitions to monitor influenza

**DOI:** 10.1186/1471-2458-12-245

**Published:** 2012-03-27

**Authors:** Matthias Nachtnebel, Benedikt Greutelaers, Gerhard Falkenhorst, Pernille Jorgensen, Manuel Dehnert, Brunhilde Schweiger, Christian Träder, Silke Buda, Tim Eckmanns, Ole Wichmann, Wiebke Hellenbrand

**Affiliations:** 1 Department of Infectious Disease Epidemiology, Robert Koch Institute, DGZ-Ring 1, Berlin 13086, Germany; 2 Post Graduate Training in Applied Epidemiology, Robert Koch Institute, Berlin, Germany; 3 European Programme for Intervention Epidemiology Training (EPIET), European Centre for Disease Prevention and Control (ECDC), Stockholm, Sweden; 4 Immunization Unit, Robert Koch Institute, Berlin, Germany; 5 National Reference Centre for Influenza, Robert Koch Institute, Berlin, Germany; 6 Vivantes Clinic, Berlin, Germany; 7 Respiratory Disease Unit, Robert Koch Institute, Berlin, Germany; 8 Surveillance Unit, Robert Koch Institute, Berlin, Germany

## Abstract

**Background:**

Surveillance of severe acute respiratory infections (SARI) in sentinel hospitals is recommended to estimate the burden of severe influenza-cases. Therefore, we monitored patients admitted with respiratory infections (RI) in 9 Berlin hospitals from 7.12.2009 to 12.12.2010 according to different case definitions (CD) and determined the proportion of cases with influenza A(H1N1)pdm09 (pH1N1). We compared the sensitivity and specificity of CD for capturing pandemic pH1N1 cases.

**Methods:**

We established an RI-surveillance restricted to adults aged ≤ 65 years within the framework of a pH1N1 vaccine effectiveness study, which required active identification of RI-cases. The hospital information-system was screened daily for newly admitted RI-patients. Nasopharyngeal swabs from consenting patients were tested by PCR for influenza-virus subtypes. Four clinical CD were compared in terms of capturing pH1N1-positives among hospitalized RI-patients by applying sensitivity and specificity analyses. The broadest case definition (CD1) was used for inclusion of RI-cases; the narrowest case definition (CD4) was identical to the SARI case definition recommended by ECDC/WHO.

**Results:**

Over the study period, we identified 1,025 RI-cases, of which 283 (28%) met the ECDC/WHO SARI case definition. The percentage of SARI-cases among internal medicine admissions decreased from 3.2% (calendar-week 50-2009) to 0.2% (week 25-2010). Of 354 patients tested by PCR, 20 (6%) were pH1N1-positive. Two case definitions narrower than CD1 but -in contrast to SARI- not requiring shortness of breath yielded the largest areas under the Receiver-Operator-Curve. Heterogeneity of proportions of patients admitted with RI between hospitals was significant.

**Conclusions:**

Comprehensive surveillance of RI cases was feasible in a network of community hospitals. In most settings, several hospitals should be included to ensure representativeness. Although misclassification resulting from failure to obtain symptoms in the hospital information-system cannot be ruled out, a high proportion of hospitalized PCR-positive pH1N1-patients (45%) did not fulfil the SARI case-definition that included shortness of breath or difficulty breathing. Thus, to assess influenza-related disease burden in hospitals, broader, alternative case definitions should be considered.

## Background

On June 11, 2009, the World Health Organization (WHO) declared an influenza pandemic caused by influenza A(H1N1)pdm09 (referred to as pH1N1 in the following). After only low levels of pH1N1 activity during spring and summer 2009, Germany registered an increase in the number of cases starting in calendar week 42 (October) 2009. The ensuing influenza wave peaked in week 46 (November) 2009 and caused almost 250 reported deaths [1] and an estimated 2.9 (95% CI: 2.5-3.4) million outpatient consultations [2].

Influenza is an acute viral disease of the respiratory tract. The majority of previously healthy individuals recover within 2 weeks. However, influenza can present as serious disease, for instance, as primary pneumonia, or lead to exacerbation of pre-existing cardiovascular and pulmonary disease. The very young, the elderly and patients with underlying illnesses are most at risk of developing these potentially life-threatening complications [3]. However, severe disease due to pH1N1 has also been observed in older children and young adults [4]. Estimates of pH1N1 case fatality range from 0.005% [5] in New Zealand, over 0.05% [6] in the United States, to 1.7% in Peru [7].

WHO and the European Centre for Disease Prevention and Control (ECDC) recommend hospital-based surveillance of severe acute respiratory infections (SARI) as a tool to monitor severe disease caused by influenza [8]. This can complement surveillance of outpatient monitoring of influenza like illness (ILI) or acute respiratory illness (ARI) to cover the full spectrum of influenza-related disease. In addition, trends in the severity of a pandemic might be detected early, and risk factors for severe disease may be identified [8,9]. A number of countries within the WHO European region have recently established SARI surveillance of different scope and profile [10].

In Germany, systems to estimate the burden of severe disease due to influenza were not well established during the 2009 influenza pandemic. One such system, the pandemic hospital based surveillance (PIKS), was implemented in week 49 in 2009 by the Robert Koch Institute [11] to collect data on patients admitted to hospitals due to laboratory confirmed influenza, their proportion among all admitted patients, influenza cases admitted to intensive care units (ICU) as well as fatal cases. However, syndromic SARI surveillance was not routinely performed.

In the winter of 2009/10 a hospital-based pandemic influenza vaccine effectiveness study was launched. This required active case finding of all admitted patients with an acute respiratory infection to identify potential influenza cases and opened a window of opportunity to monitor the number of cases admitted with respiratory infections (RI). Our objective was to monitor the epidemiology of cases admitted with RI over time in participating hospitals as the proportion of all internal medicine and ICU admissions and to determine the proportion of cases with pH1N1. We applied different case definitions (CD), including SARI, to patients admitted with RI and tested their sensitivity and specificity for detecting pH1N1 positive patients.

## Methods

### Study period

We collected data for the period from December 7, 2009 to December 12, 2010. We divided the study period into two segments according to the level of influenza activity detected in Berlin. Of all notified influenza cases in Berlin, 95% were reported before week 2 in 2010, which coincided with a decline of the German-wide consultation index of respiratory infections to background levels [12].

Elevated activity of respiratory infections was reported in week 49 (early December) 2010 and a week later, 29% of sentinel laboratory specimen sent to the Influenza Reference Centre were positive for influenza, indicating the beginning of influenza circulation in the 2010/2011 season at the end of our study period [13].

Thus, we defined study period 1 as time with significant influenza circulation which lasted from December 7, 2009 to January 10, 2010, followed by study period 2 with low or absent circulation during the spring, summer, and autumn months of 2010 (January 11, 2010 to December 12, 2010).

### Data collection

The hospital information-system (ORBIS^©^) of 9 community hospitals of the Vivantes Network for Health was screened daily for newly admitted patients. These 9 hospitals serve ~30% of the Berlin population (3.4 million inhabitants) [14]. A study team comprised of 9 study nurses and additional 12 study assistants was assigned to these locations, where they accessed electronic admission entries to identify all patients admitted with acute respiratory infections to internal medicine and ICU wards. Electronic charts were checked for symptoms fulfilling the broadest case definition (CD) for RI (defined as CD1, see below) as outlined below and for date of disease onset. In cases of incomplete documentation on admission, the study team contacted nursing staff on the respective wards to obtain this information. Moreover, the study team extracted information on age, sex, place of residence, admission to ICU, and length of hospital stay. Collected data were entered into an excel spreadsheet at the hospital and transmitted to the study centre at the Robert Koch Institute. At the study centre, data from all 9 hospitals were merged and analyzed.

The Vivantes hospital administration contributed weekly data on the aggregated numbers of all patients admitted to the hospital network in the respective periods, with separate information on admissions to internal medicine wards and ICU.

The study population was confined to patients aged 18 to 65 years, since persons older than 65 years were not included in the vaccine effectiveness study. The study protocol was approved by the Ethics Committee of the Charité- University Medicine Berlin.

### Case definitions

**Figure 1:**
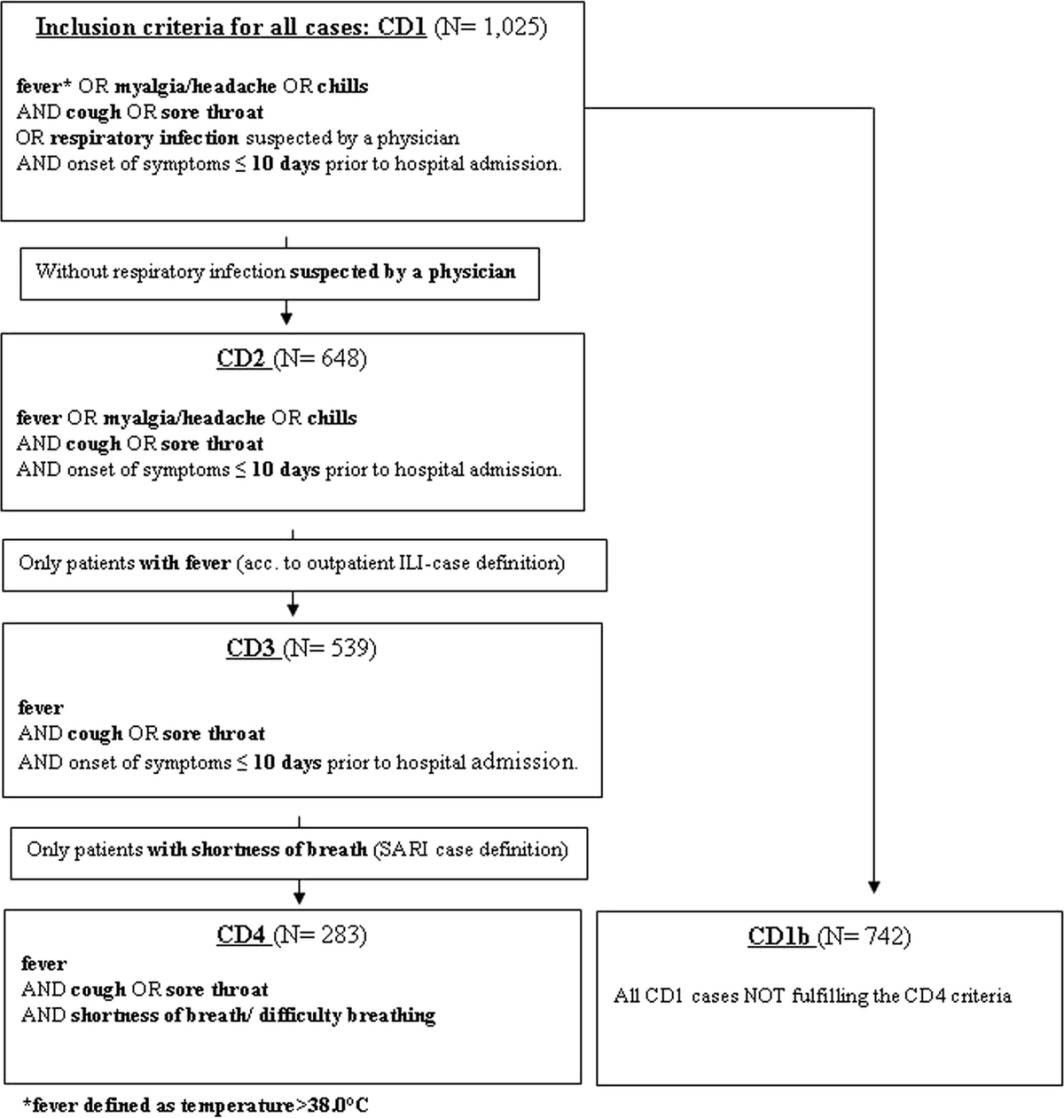
**Flowchart of case definitions (CD) for hospitalized respiratory infections**. To be included as a case in the hospital-based surveillance of acute respiratory infections in Berlin, 2009/10, patients had to fulfil criteria of the broadest case definition (CD1). All CD1 who did not meet the CD4 (SARI) criteria were classified as CD1b. CD2 and CD3 fulfilled clinical criteria only and excluded those where clinical criteria were not fully met but a physician's (suspected) diagnosis of RI in the chart led to inclusion. The smallest subsample of CD1 were cases fulfilling CD4 criteria (SARI as defined by ECDC/WHO).

We applied a broad case definition (CD1) for all hospitalized RI to enable retrospective evaluation of the sensitivity and specificity of more specific (narrower) case definitions and to capture as many pH1N1-positive cases as possible (Figure 1). CD1 thus also included patients who did not fulfil some or all of the specific clinical criteria of an RI so long as an RI was documented in the chart by a physician as the suspected diagnosis. CD4 was the narrowest case definition and was identical to the SARI case definition as suggested by ECDC [15] and WHO [8] in 2009. In addition, we tested sensitivity and specificity of two further case definitions in terms of capturing the highest proportion of pH1N1-positives among RI-patients referred to as CD2 and CD3 (Figure 1). CD2 included only those CD1 patients who fulfilled the specified clinical criteria and excluded those CD1 patients based only on a suspected diagnosis of RI. CD3 was identical to the WHO case definition for influenza like illness (ILI) [8], originally developed for the surveillance of influenza in outpatients (WHO EURO changed the ILI case definition in 2011 [16] by dropping sore throat and altering the definition of fever) (Figure 1).

Informed consent for nasopharyngeal swabbing was sought from all RI patients unless exclusion criteria as applied in the vaccine effectiveness study were present (living in a nursing home, not able to give informed consent, insufficient communication skills, contraindication for influenza vaccination). All consenting patients received a nasal and throat swab (Mastaswab; MAST Diagnostica, Reinfeld, Germany) to obtain material for real-time polymerase chain reaction (PCR) testing at the Reference Centre for Influenza. In a few patients admitted to ICU, material was collected from broncho-alveolar lavage fluids. PCR was performed as described previously, targeting the M gene for universal detection of influenza A viruses as well as the HA and NA genes for further subtyping including the specific detection of pH1N1 viruses [17]. This procedure was extensively validated. The limit of detection (95% detection probability) was determined at ~ 6 genome equivalents per reaction indicating a high sensitivity. All PCR assays used showed 100% specificity with no cross-reactivities observed with other respiratory viruses or bacteria [17].

From April 2010 onwards, we continued to register cases from all participating hospitals, but influenza diagnosis was offered only to patients from 4 hospitals covering roughly 13% of Berlin's population, resulting in a lower proportion of swabbed patients.

### Statistical methods

We calculated proportions of patients fulfilling the respective case definitions in relation to the overall number of patients admitted to internal medicine wards and ICU of the 9 hospitals. To compare medians and proportions, we used the Kruskal-Wallis and chi-square test, respectively. The Breslow-Day test was used to assess homogeneity between strata. As measures of association we calculated relative risks (RR) as well as odds ratios (OR) and their 95% confidence intervals (CI) by univariable exact logistic regression. To evaluate trends over time we applied Poisson regression and calculated incidence rate ratios (IRR) and 95% CI of the mean. As a measure of comparison for the different case definitions we utilized the area under the receiver operator curves (ROC) and 95% CI (asymptotic normal) of the area under the curve (AUC); we compared AUC with chi-square tests. Cases testing negative were only included as such if the nasopharyngeal swab was taken within 7 days of symptom onset. A sensitivity analysis was performed using a swabbing interval of 4 days. Data analysis was performed with Stata^©^, version 11.

## Results

### Number of cases according to case definition and severity

Overall, we identified 1,025 patients fulfilling the broad CD1 definition (Table 1). Of these, 283 (28%) fulfilled CD4 criteria. The remaining 742 (72%) cases were classified as CD1b. Case definition criteria for CD2 were met by 63% (n = 648) and for CD3 by 53% (n = 539).

**Table 1:** Patients with respiratory infections according to case definition and pH1N1 status, demographic and clinical characteristics, hospital-based surveillance of acute respiratory infections, Berlin 2009/10

Cases Definition (number of cases)	% male	Median age, females*	Median age, males	% requiring ICU treatment	% requiring mechanical ventilation	% pH1N1 positive among tested	Duration of hospital stay (median)	Case fatality
**CD1 **(n = 1,025)	61%*	49 y	51 y	12%	6%	6%	6 days	4%

**CD1b **(n = 742)	62%*	50 y	51 y	11%	5%	4%	6 days	4%

**CD4 **(n = 283)	57%*	45 y	50 y*	14%	7%	7%	7 days	2%

**pH1N1-positive **(n = 20)	65%	42 y	46 y	20%	7%	100%	6 days	0%

* statistically significant (*p *< 0.05) when compared to female cases

See text and Figure 1 for explanation of CD1, 1b and 4

We tested 354 (35%) patients for influenza virus infection. From calendar week 50/2009 to 14/2010, 291 ARI patients were identified in the 9 participating hospitals, of whom 141 (48%) agreed to swabbing. From calendar week 15/2010 to 49/2010, 734 ARI patients were ascertained, of whom 365 (50%) were from the 4 hospitals in which testing continued to be offered. Of these, 196 (54%) agreed to provide a nasopharyngeal swab. Reasons for refusal were not systematically documented, but there was no significant difference in age (*p *= 0.3) or gender distribution (*p *= 0.2) between those tested or not. In study period 1, 16 of 56 cases with test results available (29%) tested pH1N1-positive versus 4 of 286 (1%) in period 2 (Table 2). Overall, CD4 patients were more likely to give informed consent for nasopharyngeal swabbing than CD1b patients (RR = 1.9, 95%CI: 1.60-2.21). The proportion of pH1N1-positives did not differ significantly between those CD4 and CD1b cases (*p *= 0.3) that were tested, neither during period 1 (32% vs. 25%, *p *= 0.5) nor period 2 (2% vs. 1%, *p *= 0.7).

**Table 2:** Absolute and relative number of patients tested positive for pH1N1 by case definition and study period, hospital-based surveillance of acute respiratory infections, Berlin, 2009/10

		No. tested for influenza (%)	No. testing positive for influenza* (%)
**CD1**	Period 1	60/110 (55%)	16 (29%)
	
	Period 2	294/915 (32%)	4 (1%)
	
	Total	354/1,025 (35%)	20 (6%)

**CD1b**	Period 1	32/71 (45%)	7 (25%)
	
	Period 2	174/671 (26%)	2 (1%)
	
	Total	206/742 (28%)	9 (5%)

**CD4**	Period 1	28/39 (72%)	9 (32%)
	
	Period 2	120/244 (49%)	2 (2%)
	
	Total	148/283 (52%)	11 (8%)

*Patients with missing PCR results excluded

A higher proportion of both CD1 cases and pH1N1-positive patients was male (Table 1). Among CD4 cases men were older than women (*p *= 0.04), but there was no statistically significant difference between the age of men and women fulfilling the CD1 or the CD1b criteria (*p *= 0.7). The median age of CD4 patients was lower than that of CD1b patients (*p *= 0.05). Exact logistic regression, however, did not reveal a dose-response relationship between years of age and odds of fulfilling the CD4 definition (*p *= 0.2). Median age of pH1N1-positives was lower than in negatives but this was not statistically significant (*p *= 0.2).

Of the 20 pH1N1- positive cases, 55% were classified as CD4. Only four pH1N1-positive cases (20%) were admitted to ICU, of which 3 (75%) were classified as CD4. None of the 20 pH1N1-positive cases died, but pH1N1-status was positively associated with ICU-admission, although this was not statistically significant (*p *= 0.09). Neither requiring mechanical ventilation (*p *= 0.8) nor length of hospital stay (*p *= 0.6, Table 1) showed an association with pH1N1-status. The risk of ICU-admission (RR = 1.3, 95%CI: 0.95-1.93) and of requiring mechanical ventilation (RR = 1.3, 95%CI: 0.69-2.41) was similar for CD1b and CD4 cases, but median duration of hospital stay was longer for CD4 than CD1b cases (*p *= 0.03). Death during hospital admission occurred in 2% of CD4 and 4% of CD1b cases (*p *= 0.13), but data on patient outcome was missing in 23% of all included cases. The proportion of cases with missing patient outcome data did not differ by pH1N1-status, admission to ICU or CD4 versus CD1 (all p-values > 0.1).

The likelihood of hospitalized RI-patients fulfilling criteria for the narrowest case definition (CD4) was 1.3 times higher (95%CI: 1.01-1.75) in study period 1 than period 2 (35% versus 27%). In addition, CD1-cases had a RR of 1.7 (95%CI: 1.07-2.62) for admission to ICU and of 2.6 (95%CI: 1.31-5.04) for requiring mechanical ventilation during period 1 compared to period 2.

Overall, 185 of 353 (52%) influenza tests were performed more than 4 days after onset of disease and 80 of 353 (23%) more than 7 days. During study period 1, none of the 16 pH1N1-positive cases was swabbed > 7 days after symptom onset versus 11 of 40 (28%) pH1N1-negatives (OR = 0.13; 95%CI: 0-0.85; *p *= 0.03). Four (25%) pH1N1-positive cases, but 24 of 40 (60%) pH1N1-negative case were swabbed > 4 days (p = 0.018) after symptom onset.

### Differences between hospitals

Absolute and relative numbers of patients admitted due to RI varied by study location. Over the entire study period, the mean proportion of CD1-cases among internal medical ward admissions was 4.9% and ranged from 3.3% to 8.4% between hospitals (*p *< 0.01). The proportion of CD4 cases among all respiratory infections was 28% overall and ranged from 19% to 44% in participating hospitals (*p *< 0.01). These results indicated heterogeneity among study locations. Neither the percentage of pH1N1 positives nor the gender distribution differed significantly by hospital among those tested (*p *= 0.2), nor did the gender distribution (*p *= 0.12). However, median age of included RI-patients ranged from 45 to 56 years by study location (*p *< 0.01).

### Development over time

**Figure 2:**
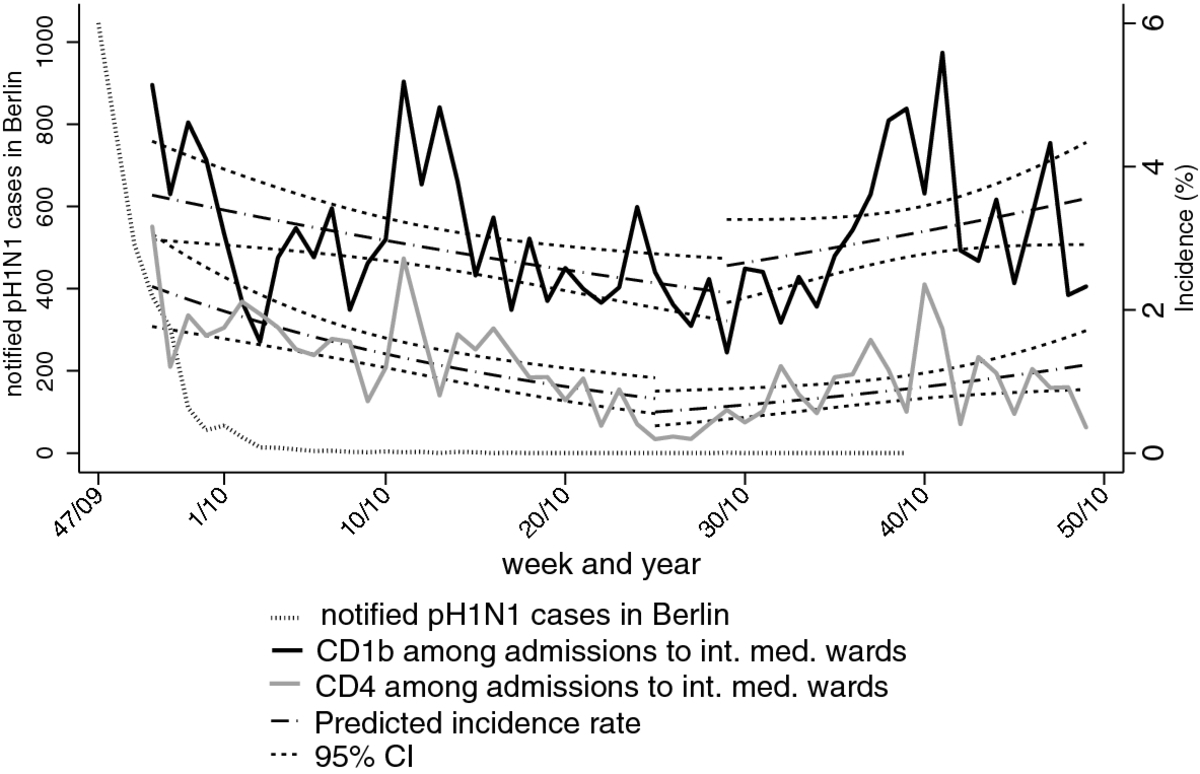
**Incidence of hospitalized respiratory infections fulfilling criteria for CD1b and CD4 as a percentage of internal medicine ward admissions in the study hospitals per calendar week**. Weekly incidences are shown together with predicted incidences and 95%CI according to Poisson regression. All pH1N1 cases notified in Berlin through the national routine surveillance system, 2009-2010 are shown for comparison.

**Figure 3:**
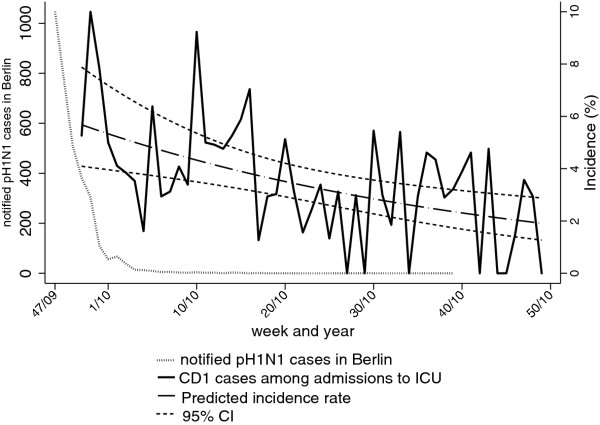
**Incidence of respiratory infections fulfilling criteria for CD1 admitted to intensive care units (ICU) as a percentage of all admissions to ICU per calendar week**. Weekly incidences are shown together with predicted incidences and 95%CI according to Poisson regression. All pH1N1 cases notified in Berlin through the national routine surveillance system, 2009-2010 are shown for comparison.

We calculated the weekly incidence of all CD1-cases, CD1b cases, and CD4 cases as a percentage of all admissions to internal medicine wards and of all CD1-cases admitted to ICU as a percentage of all ICU admissions (Figure 2 and 3, Table 3). All incidences showed statistically significant changes over time. Admission of CD1b cases exhibited a biphasic pattern with a decline from 5.1% in week 50/2009 to a minimum of 1.4% in week 29/2010, with a weekly IRR of 0.99 (95%CI: 0.98-1.00). Thereafter, the IRR was 1.02 (95%CI: 1.00-1.03) per week until the end of the study period with a maximum of 5.6% in week 41 in 2010. We observed a similar pattern for CD4 cases with an IRR of 0.96 (95%CI: 0.94-0.98) per week to a minimum of 0.2% in week 25 in 2010; followed by a weekly IRR of 1.03 (95%CI: 1.00-1.06). In contrast, the incidence of all cases among ICU admissions decreased over the entire study period with an IRR of 0.98 per week (95%CI: 0.97-0.99).

**Table 3:** Minimum and maximum weekly incidence of respiratory infections as a proportion of internal medicine admissions and intensive care unit (ICU) admissions, respectively, according to case definition (CD), hospital-based surveillance of acute respiratory infections, Berlin, 2009/10

Case definition	Maximum	Week	Minimum	Week
**CD1 among internal medicine admissions**	8.3%	50/2009	2.0%	27/2010

**CD1b among internal medicine admissions**	5.6%	41/2010	1.4%	29/2010

**CD4 among internal medicine admissions**	3.2%	50/2009	0.2%	25/2010

**CD1 cases among ICU-admissions**	10.0%	51/2009	0%	repeatedly

See text and Figure 1 for explanation of CD1, 1b and 4

### Sensitivity and specificity of case definitions

The sensitivity and specificity of the case definitions applied to all included patients using results of PCR testing as the gold standard are shown in Table 4 both for the entire study period and separately for study period 1 with significant influenza virus circulation. The highest sensitivity was found for CD2; the highest specificity for CD4.

**Table 4:** Comparison of case definitions (CD) for hospitalized respiratory infections (RI) according to their ability to capture pH1N1-positive cases

	Case definition	Cases from entire study period			Cases from study period 1		
		
		Sensitivity	Specificity	Area under ROC (95% CI)	Sensitivity	Specificity	Area under ROC (95% CI)
**Swabbing interval ≤ 4 days**	**CD4**	55%	**59%**	0.57 (0.45-0.69)	56%	**63%**	0.59 (0.42-0.77)

	**CD2**	**95%**	26%	0.60 (0.54-0.66)	**100%**	44%	**0.72** **(0.59**-**0.84)**

	**CD3**	90%	41%	**0.65** **(0.57**-**0.73)**	94%	44%	0.69 (0.55-0.83)

**Swabbing interval ≤ 7 days**	**CD4**	55%	**57%**	0.56 (0.45-0.68)	56%	**59%**	0.57 (0.42-0.73)

	**CD2**	**95%**	22%	0.59 (0.53-0.64)	**100%**	38%	**0.69** **(0.60**-**0.78)**

	**CD3**	90%	35%	**0.63** **(0.55**-**0.70)**	94%	41%	0.68 (0.57-0.79)

Sensitivity, specificity, area under the receiver operator curve (ROC), percentage of pH1N1-positives captured for study period 1 (significant influenza virus circulation in Berlin) and the entire study period (gold standard: PCR confirmation of pH1N1 infections)

*Cases testing negative were only included as pH1N1-negative if the interval from symptom onset to swabbing was ≤ 4 days or ≤ 7 days, respectively. Highest ROC area in bold.

**Figure 4:**
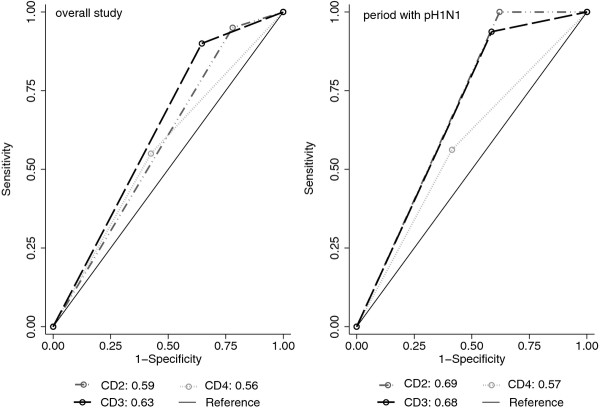
**Receiver Operator Curve (ROC) comparing the capability of different case definitions (CD) for hospitalized respiratory infections (RI) to capture cases of influenza A/H1N1 2009 (pH1N1)**. Areas under the ROC curve for CD2, CD3 and CD4 using pH1N1 PCR results as the gold standard for the overall study period (left) and the period with significant pH1N1 in Berlin (right). Data based on cases of respiratory infections hospitalized in the participating hospitals and tested for influenza, Berlin, December 2009 to December 2010. pH1N1 negative cases included only if period between symptom onset and nasopharyngeal swab ≤ 7 days.

The area under the ROC curve was largest for CD2 in study period 1 and for CD3 during the entire study period, although not statistically significantly larger than for CD4 (*p *> 0.1) (Table 4, Figure 4). ROC areas increased only minimally if a maximal swabbing interval of 4 instead of 7 days was applied to ascertain pH1N1 negatives.

## Discussion

The implementation of a surveillance system monitoring respiratory diseases at 9 Berlin hospitals initially in the context of a hospital-based vaccine effectiveness study was feasible and delivered important insights into the distribution of cases over time and according to different CD. A large proportion of pH1N1 positives was not captured by the SARI case definition as recommended by WHO and ECDC (CD4), with alternative case definitions having a better combination of sensitivity and specificity (Table 4).

As expected, the incidence of both CD1b and CD4-cases was subject to the influence of seasonality. The proportion of cases fulfilling the narrowest CD4 definition was highest during study period 1 (winter), a period with substantial influenza virus circulation, and significantly lower thereafter. This was also reflected in the higher proportion of RI patients admitted to ICU during period 1. These findings are in keeping with a seasonal distribution of RI as described previously [18-20]. Our results suggest that the spectrum of pathogens that circulate during winter causes a higher proportion of severe cases than the pathogen mix prevailing in spring and summer. Besides influenza, many respiratory pathogens exhibit seasonal patterns with peaks during the winter, e.g. adeno- or coronavirus infections [21], but our data cannot provide insight as to which individual pathogens might cause increased morbidity. In our study almost a third of all RI-patients was positive for influenza during study period 1, and other studies have shown that influenza viruses are among the most common pathogens leading to hospitalization [21-23] for respiratory disease or cardio-respiratory failure in the winter season [24]. Nonetheless, a significant proportion of respiratory disease is apparently caused by other pathogens; even if possible underestimation of the proportion of influenza due to late swabbing in our study is taken into account.

We found that 20% of all hospitalized pH1N1-positive patients required ICU treatment, a number within the range of previously reported figures for pH1N1 illness [25,26]. The higher risk of admission to ICU, mechanical ventilation and death in CD4 compared to CD1b cases was not statistically significant; this may have been due to insufficient power. Hospital stay was significantly longer in CD4 than in CD1b-cases.

The definition of CD4 (identical to the WHO/ECDC SARI case definition) captured only 55% of all hospitalized pH1N1-positive cases, and 75% of the pH1N1-cases requiring ICU management. Although a higher proportion of CD4 than CD1b-cases was pH1N1-positive, this difference was not statistically significant in either study period. Thus, we compared sensitivity and specificity of different case definitions to explore the ability of varying combinations of symptoms to capture PCR-confirmed pH1N1-influenza (gold standard) within our study population of hospitalized RI-patients. CD2, which permitted other systemic symptoms as an alternative to fever and did not require shortness of breath (SOB), had the highest sensitivity. The sensitivity of CD3, which differed from CD2 only in the strict requirement for fever, was only slightly lower. CD4 (SARI) had the lowest sensitivity. The trade-off for high sensitivity was a low specificity, which applied in reverse order to the tested case definitions. By comparing the areas under the ROC curves we took both measures -sensitivity and specificity- into account and found comparable and most favourable results for CD2 and CD3, at least during the period with significant influenza circulation. However, although the area under the ROC was highest for CD4, the difference to CD2 and CD3 was not statistically significant, likely due to overall low case numbers related to the rather late start of our study towards the end of the pandemic wave. The optimal syndromic case definition for respiratory infections to monitor severe (hospitalized) influenza cases has been subject to repeated discussions as, for instance, the 2011 update to the ILI (CD3) case definition by WHO Europe highlights: sore throat as a symptom was dropped and a history of fever as an additional qualifying symptom added [16]. We were not able to assess the latter case definition as information on the presence of sore throat was not documented separately from cough in our study. However, our findings suggest that the broader case definitions offer advantages for surveillance of hospitalized influenza cases over the current SARI case definition as suggested by WHO/ECDC.

Hospitals showed a significant heterogeneity with respect to the proportions of patients admitted with RI. This suggests multiple study sites should be carefully selected to ensure representative surveillance of respiratory disease. Since we were interested in the trend of RI on the level of Berlin's population rather than single hospitals we did not stratify by hospital location.

Our study had some important limitations. The link to the vaccine effectiveness study led to a rather late start of our hospital-based surveillance, 2-3 weeks after the peak of the pH1N1-pandemic in Berlin and just a few weeks before it subsided. This resulted in the inclusion of a rather small number of pH1N1-positive patients. More comprehensive results would also have been possible if children and patients older than 65 years had been included in the study. Furthermore, the study ended just at the start of the 2010/2011 influenza season. Nonetheless, conduction of SARI-surveillance was found to be feasible in the framework of a hospital-based vaccine effectiveness study and can be considered in future pandemic situations if early implementation is possible. At least in our setting, the presence of trained study nurses was essential for the success of SARI-surveillance due to the extra work required to extract relevant information from the hospital information-system.

Almost a quarter (23%) of nasal and throat swabs was taken more than 7 days after symptom onset. This proportion differed by pH1N1-status: 5% among pH1N1-positives versus 24% among negatives (*p *= 0.052), suggesting potential misclassification of pH1N1-positives as negative. However, other studies reported shedding of influenza virus for 6 to 9 days and, moreover, that shedding in hospitalized patients and with underlying illness appears to be longer than in households [27-32]. In addition, we cannot rule out misclassification from inability to obtain or document symptoms in the hospital information-system. This could also be an explanation for the lower sensitivity of the CD4 case definition. Clinical information on underlying disease, which might serve to explain gender differences in regards of admission to ICU, was only available for patients included in the vaccine effectiveness study (results not shown), but was not available for all patients in the SARI-surveillance. Finally, our study was confined to one large city and is therefore not necessarily representative for other populations.

## Conclusions

Overall, experience with SARI-surveillance is scarce and urgently needed to establish the best practise of monitoring disease burden due to severe influenza. Although our surveillance began after the peak of the pandemic, our results permitted the depiction of decreasing influenza activity at the end of the pandemic wave. Our study added valuable insights, such as into the overall burden of RI patients requiring hospitalization, ICU admission and mechanical ventilation in periods with and without influenza virus circulation. In addition, our ROC comparison suggested that slightly broader case definitions than the WHO/ECDC SARI definition can capture a higher proportion of pH1N1 positive cases and might therefore be more appropriate for surveillance of hospitalized influenza during epidemic waves or a pandemic.

## Competing interests

The authors declare that they have no competing interests.

## Authors' contributions

MN drafted the manuscript, was involved in the data collection process, and was responsible for the data analysis and interpretation. BG, GF, PJ, CT, OW, and WH were involved in the study design, data collection, and review of the draft manuscript. MD, TE, and SB had made significant contributions to the analytical part, interpretation of results, and review of the manuscript. BS was responsible for the laboratory testing of specimen, interpretation of laboratory results, and reviewed the draft manuscript. All authors read and approved the final manuscript.

## Pre-publication history

The pre-publication history for this paper can be accessed here:

http://www.biomedcentral.com/1471-2458/12/245/prepub
